# Targeting SREBP-1-Mediated Lipogenesis as Potential Strategies for Cancer

**DOI:** 10.3389/fonc.2022.952371

**Published:** 2022-07-14

**Authors:** Qiushi Zhao, Xingyu Lin, Guan Wang

**Affiliations:** ^1^ National Engineering Laboratory for AIDS Vaccine, Key Laboratory for Molecular Enzymology and Engineering, The Ministry of Education, School of Life Sciences, Jilin University, Changchun, China; ^2^ Department of Thoracic Surgery, The First Hospital of Jilin University, Changchun, China

**Keywords:** SREBP-1, fatty acid synthase, fatty acids, lipogenesis, cancer therapy

## Abstract

Sterol regulatory element binding protein-1 (SREBP-1), a transcription factor with a basic helix–loop–helix leucine zipper, has two isoforms, SREBP-1a and SREBP-1c, derived from the same gene for regulating the genes of lipogenesis, including acetyl-CoA carboxylase, fatty acid synthase, and stearoyl-CoA desaturase. Importantly, SREBP-1 participates in metabolic reprogramming of various cancers and has been a biomarker for the prognosis or drug efficacy for the patients with cancer. In this review, we first introduced the structure, activation, and key upstream signaling pathway of SREBP-1. Then, the potential targets and molecular mechanisms of SREBP-1-regulated lipogenesis in various types of cancer, such as colorectal, prostate, breast, and hepatocellular cancer, were summarized. We also discussed potential therapies targeting the SREBP-1-regulated pathway by small molecules, natural products, or the extracts of herbs against tumor progression. This review could provide new insights in understanding advanced findings about SREBP-1-mediated lipogenesis in cancer and its potential as a target for cancer therapeutics.

## Introduction

Sterol regulatory element-binding proteins (SREBPs) are identified as a family of transcription factors with the domain of a basic helix–loop–helix leucine zipper (bHLH-LZ), which regulate genes involved in the pathways of lipid synthesis and uptake (1–3). In mammalian cells, three SREBP isoforms, SREBP-1a, SREBP-1c, and SREBP-2, from two genes, *SREBF1* and *SREBF2*, have been identified, which have overlapping transcriptional programs for the synthesis of fatty acids and cholesterol (4, 5). SREBP-1a and SREBP-1c are derived from the same gene through alternative splicing at transcription start sites (6), but they have different regulations for downstream target genes (4, 7). A study conducted for about 30 years has confirmed that SREBP-1c is involved in regulating fatty acid synthesis and lipogenesis and SREBP-1a can be implicated in two pathways of SREBP-1c and SREBP-2 (specific to cholesterol metabolism) (8). Under pathological conditions, SREBP-1 activation can cause lipid dysfunction to contribute to various metabolic diseases, such as obesity, diabetes mellitus, non-alcoholic fatty liver disease, and cancer (9–12). Recently, more and more evidence has demonstrated that SREBP-1 participates in metabolic reprogramming in different cancer, such as prostate cancer (13), breast cancer (14), and glioblastoma (GBM) (15), which has been a potential target for cancer therapy. Moreover, several essential pathways, such as epidermal growth factor receptor (EGFR), phosphatidylinositol 3-kinase (PI3K)/protein kinase B (PKB, Akt)/mammalian target of rapamycin (mTOR), and Ras, can regulate SREBP-1 activation to mediate tumor growth and metastasis (15, 16). Importantly, multiple treatment strategies targeting the SREBP-1 signaling pathway, including small molecules or genetic inhibition, have been extensively studied and developed (17, 18). The preparation of the publications in this review was conducted as follows: 1) the articles in English were electronically searched from October 1993 to May 2022 in the databases of PubMed and the Web of Science. 2) “SREBP-1” or “sterol regulatory-element binding protein-1” and “cancer” were used as the search terms. 3) A secondary search was performed by checking the title and the abstract to collect published papers with the inclusion criteria. In this review, we summarized the key upstream signaling pathway and the function of SREBP-1-regulated lipogenesis in different cancer. We also discussed potential therapies targeting the SREBP-1-regulated pathway against tumor progression. This review could provide new insights in understanding advanced findings about SREBP-1-mediated lipid dysfunction in cancer and its potential as a target for cancer therapeutics.

## SREBP-1 activation and its downstream targets in cancer

Human gene *SREBF1* (26 kb, 22 exons, and 20 introns) at a chromosomal location of 17p11.2 was cloned and characterized in 1995, as a result of alternative splicing at both the 5′ and 3′ ends (19, 20). SREBP-1a and SREBP-1c differ in the extreme N-terminal acidic amino acids, which share a similar structure containing an NH_2_-terminal transcription factor domain (480 amino acids), a middle hydrophobic region (80 amino acids), and a COOH-terminal regulatory domain (590 amino acids) (3). SREBP-1a and SREBP-1c include 42 amino acids (12 acidic acids) and 24 amino acids (six acidic acids) in the acidic NH_2_-terminal domain, respectively. SREBP-1c has a much weaker effect in transcription activation than that of SREBP-1a, due to its shortened acidic domain (21). SREBP-1 is conserved from fission yeast to humans (22), and its two isoforms are found in the liver, adipose, and skeletal muscle tissues (20, 23, 24). SREBP-1c is more abundant than SREBP-1a in the liver (21, 25), and SREBP-1a is abundantly expressed in some tissues and cells, such as heart, macrophages, and dendritic cells from bone marrow (26). Recently, it has been revealed that SREBP-1 is obviously activated in different cancers, which is higher than that of non-tumor tissues (13, 27) and has been a biomarker for the prognosis or drug efficacy for patients with cancer (14, 28).

**Figure 1:**
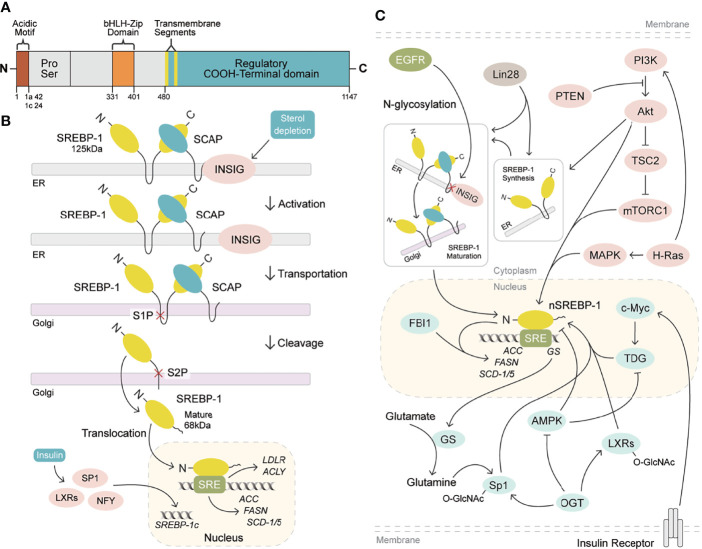
SREBP-1 structure, activation, and signaling pathways. **(A)** SREBP-1 structure and activation. SREBP-1 contains the NH2-terminal domain (the bHLH-Zip motif and an acidic motif), a middle hydrophilic region, and the COOH-terminal domain. **(B)** After INSIG dissociation from SCAP, SREBP-1 translocates to the Golgi apparatus and is cleaved by site 1 protease (S1P) and site 2 protease (S2P) to form a nuclear form (nSREBP-1) for activating the transcription of its downstream targets, such as FASN, ACC, SCD-1/5, ACLY, and LDLR. **(C)** Multiple signaling pathways regulate SREBP-1 expression, translocation, and maturation, including EGFR, PI3K/Akt/mTORC1, and others. OGT: O-GlcNAc transferase, GS: glutamate synthetase, TDG: thymine DNA glycosylase.

SREBP-1a and SREBP-1c are synthesized as 125-kDa precursors in endoplasmic reticulum membrane and cleaved into an NH_2_-terminal fragment (68 kDa) by site 1 and site 2 proteases to translocate into the nucleus for lipogenesis gene transcription, when kept in the environment from sterol depletion (29–31). SREBP-1 can form a complex with SREBP cleavage-activating protein (SCAP) to mediate its cleavage in sterol depletion or in the nutritional environment (10, 25). Insulin increase by carbohydrate ingestion can cause the decrease of Insig-2a, which leads to the release of the SREBP-1c/SCAP complex to stimulate SREBP-1c cleavage in the Golgi for the activation of lipogenic/glycolytic genes (25, 32). Moreover, insulin can increase SREBP-1c expression by activating the promoters containing the binding sites for the liver X receptor, Sp1, nuclear factor-Y, and SREBP-1c (33). In addition, the cleavage of SREBP-1c is regulated by polyunsaturated fatty acids, not as SREBP-2 by SCAP or Insig-1 (34). After the cleavage of SREBP-1, the nuclear form of SREBP-1 binds to sterol regulatory elements (SREs) to stimulate the transcriptions of lipogenic genes (25, 29). As reported, SREBP-1 can activate a series of genes for fatty acid synthesis, including ATP citrate lyase (ACLY), acetyl-CoA carboxylase (ACC), fatty acid synthase (FASN), and stearoyl-CoA desaturase 1 and 2 (rodent)/5 (human) (SCD-1 and -2/5), and for lipid uptake, such as low-density lipoprotein receptor (LDLR) (4, 30–34). Furthermore, it has been reported that SREBP-1 activation can be involved in various cellular functions, such as cell proliferation, cell cycle, muscle cell differentiation, foam cell formation, and somatic cell reprogramming (35–39). Critically, the activation of SREBP-1 obviously stimulates *de novo* lipogenesis to regulate cell growth, cell cycle progression, and metastatic characteristics for cancer progression (12, 13, 40–42). Meanwhile, these genes regulated by SREBP-1, including ACLY, ACC, FASN, and SCD, are highly expressed in cancer tissues compared to adjacent non-tumor tissues, which play essential roles in the growth, metastasis, and survival of various cancers (43–46). Taken together, SREBP-1 and its target genes are essential in cancer progression, which have been potential targets for pharmacological or genetic therapy (**Figures 1A, B** **)**.

## SREBP-1-regulated lipogenesis in cancers

Currently, multiple signaling pathways control SREBP-1 activation to regulate the downstream genes for lipogenesis, including the EGFR, PI3K/Akt/mTOR, and p53 mutation pathways. EGFR signaling promotes N-glycosylation of SCAP to reduce the association with Insig-1, which consequently induces the proteolytic activation of SREBP-1 (47). Importantly, the hyperactive mutation of PI3K/Akt/mTOR signaling can inhibit oxidative stress and ferroptotic death by regulating SREBP-1/SCD-1-mediated lipogenesis (48). Akt activation can induce the synthesis of full-length SREBP-1 and cause the accumulation of nuclear SREBP-1 to increase the accumulation of intracellular lipids, such as fatty acids and phosphoglycerides, which is sterol sensitive for activating the FASN promoter (16). The mammalian/mechanistic target of rapamycin complex 1 (mTORC1) regulates the transcription and translation of SREBP-1 and SREBP-2 to promote *de novo* lipid biosynthesis in response to nutrients or insulin, which regulate cell proliferation, mitochondrial metabolism, or glycolytic capacity in cancer (49, 50). Similarly, insulin/glutamine deprivation activates SREBP-1 to bind to the promoter of glutamine synthetase (GS) for its transcription. GS results in the O-linked N-acetylglucosaminylation (O-GlcNAcylation) of specificity protein 1 (Sp1) for the induction of the SREBP-1/ACC1 pathway, which increases lipogenesis and lipid droplet accumulation in liver and breast cancer (51). Based on the key role of O-GlcNAcylation, it has been found that O-GlcNAc transferase (OGT) regulates SREBP-1 expression and its targets in a proteasomal and AMP-activated protein kinase (AMPK)-dependent manner (52). Meanwhile, OGT increases the O-GlcNAcylation of liver X receptors to induce the expression of secretory clusterin (sCLU), which is closely associated with SREBP-1c and its mediated activation of the CLU promoter for the contribution of cisplatin resistance (53). In addition, insulin can activate c-Myc to promote the transactivation of thymine DNA glycosylase (TDG), which decreases the abundance of 5-carboxylcytosine in the SREBP-1 promoter for the upregulation of SREBP-1 and ACC1. On the other hand, AMPK can inhibit DNA demethylation of the SREBP-1 promoter mediated by TDG, suggesting that TDG regulated by insulin or metformin is a potential therapeutic target for T_2_DM-related cancers (54). In the tumor immune microenvironment, Treg cells maintain the metabolic fitness and mitochondrial integrity of M2-like tumor-associated macrophages indirectly by repressing IFNγ production derived from CD8^+^ T cells, which is dependent on SREBP-1 and its mediated fatty acid synthesis (55). Under the conditions of serum starvation and hypoxia, cholesterol or O_2_ deficiency mediates the expression of the FVII gene encoding coagulation factor VII *via* the SREBP-1/glucocorticoid-induced leucine zipper, which leads to the production and shedding of procoagulant extracellular vesicles (56). A tumor gene, H-ras can upregulate mitogen-activated protein (MAP) kinase and PI3K signaling, which increases SREBP-1 and FASN levels to drive mammary epithelial cell malignant transformation and tumor virulence (57). A proto-oncogenic transcription factor, FBI-1 can directly interact with SREBP-1 *via* DNA-binding domains to synergistically activate FASN transcription for maintaining rapid cancer cell proliferation (58). An RNA-binding protein, Lin28 enhances SREBP-1 translation and maturation to promote cancer progression by directly binding to the mRNAs of both SREBP-1 and SCAP (59). Taken together, EGFR, PI3K/Akt, and mTOR signal pathways can control SREBP-1 expression and activation to regulate its downstream pathways in cancers **(** **Figure 1C**, **Table 1** **).**

**Table 1:** Main key pathways regulate SREBP-1 activation for lipogenesis.

Key upstream signal pathways	Molecular mechanism	Refs
EGFR	Promotes SCAP N-glycosylation to reduce the interaction of Insig-1 for SREBP-1 activation	(47)
Mutation of PI3K/Akt/mTOR	Inhibits oxidative stress and ferroptotic death by regulating the SREBP-1/SCD-1 pathway	(48)
Akt	Induces full-length SREBP-1 synthesis and causes nuclear SREBP-1 accumulation for increased intracellular lipid	(16)
mTORC1	Activates SREBP-1 transcription to regulate lipid synthesis *in vitro*	(49)
mTORC1	Regulates the translation and transcription of PGC-1α, SREBP-1/2, and HIF-1α and the translation of nucleus-encoded mitochondrial mRNAs	(50)
Glutamine synthetase	Causes the O-linked N-acetylglucosaminylation of the specificity protein 1 to induce the SREBP-1/ACC1 pathway for increased lipogenesis under the conditions of insulin/glutamine deprivation-induced SREBP-1 activation	(51)
O-GlcNAc transferase	Regulates SREBP-1 expression and its transcriptional targets, FASN, ACLY, ACC, and lipoprotein lipase *via* AMPK	(52)
O-GlcNAc transferase	Enhances secretory clusterin expression *via* liver X receptors and SREBP-1 for regulating cell cycle, apoptosis, and cisplatin resistance	(53)
Insulin	Upregulates c-Myc, SREBP-1, and ACC1 expression to transactivate thymine DNA glycosylase for the decrease of 5-carboxylcytosine abundance in the SREBP-1 promoter	(54)
Treg cells	Promotes SREBP-1-mediated lipid metabolism of M2-like tumor-associated macrophages by suppressing interferon-γ secretion from CD^8+^ T cells	(55)
Cholesterol starvation and hypoxia	Mediates coagulation factor VII expression by regulating the SREBP-1/glucocorticoid-induced leucine zipper pathway	(56)
H-ras	Upregulates MAPK and PI3K signaling to increase the levels of SREBP-1 and FASN for driving malignant transformation and tumor virulence	(57)
FBI-1	Directly interacts with the DNA-binding domains of SREBP-1 to activate FASN transcription for rapid cancer cell proliferation	(58)
Lin28	Enhances the translation and maturation of SREBP-1 by directly binding the mRNAs of both SREBP-1 and SCAP for cancer progression	(59)

## Colorectal cancer

The SREBP-1 expression in colorectal cancer (CRC) tissues is significantly higher than that in non-cancerous or normal tissues (60, 61). The colocalization of SREBP-1 and FASN is found in CRC tissues, and the transcriptional regulation of SREBP-1 on the FASN promoter can respond to the deficiency in endogenous fatty acid synthesis in colorectal neoplasia (62). Moreover, the combination of SREBP-1 and ACLY can significantly promote CRC cell proliferation, migration, and invasion, which is mediated by lipid synthesis modulation (63). In addition, the overexpression of SREBP-1 promotes the angiogenesis and invasion of CRC cells by promoting MMP-7 expression related to NF-κB p65 phosphorylation (61). SREBP-1 silencing decreases the levels of fatty acid and SREBP-1 targets genes to inhibit the initiation and tumor progression of CRC, which might be associated with the alternation of cellular metabolism, including mitochondrial respiration, glycolysis, and fatty acid oxidation (64). SREBP-1 also can interact with c-MYC to enhance its binding ability to the snail family transcriptional repressor 1 (SNAIL) promoter to regulate its expression and promote epithelial–mesenchymal transition in CRC (65). For the resistance of chemotherapy in CRC, SREBP-1 is overexpressed in chemoresistant CRC samples and is correlated with poor survival. SREBP-1 can increase the chemosensitivity to gemcitabine through downregulation of caspase-7, which might be a new prognostic biomarker of CRC (66). Meanwhile, the overexpression of SREBP-1 enhances the resistance of 5-FU through regulation of caspase-7-dependent PARP1 cleavage in CRC (60). On the other hand, radiation stimuli can rapidly increase SREBP-1/FASN signaling to cause cholesterol accumulation, cell proliferation, and cell death, suggesting that targeting the SREBP-1/FASN/cholesterol axis is a potential strategy for CRC patients undergoing radiotherapy (67). Recently, results from colon cancer cells and xenograft tumor models have demonstrated that RAS protein activator-like 1 inhibits colon cancer cell proliferation by regulating LXRα/SREBP1c-mediated SCD1 activity (68). An lncRNA ZFAS1 can bind polyadenylate-binding protein 2 to stabilize and increase the levels of SREBP-1 and its targets, FASN and SCD1, for the promotion of lipid accumulation in CRC (69). In rapamycin-resistant colon cancer cells, diacylglycerol kinase zeta can promote mTORC1 activation and cell-cycle progression, which are essential for SREBP-1 expression (70). Together, these findings demonstrate that SREBP-1 participates in colon cancer growth, invasion, and the resistance of chemotherapy or radiation, which have been potential therapeutic targets for CRC treatment.

## Prostate cancer

During the castrated progression of a prostate cancer xenograft model, SREBP-1a and SREBP-1c are significantly greater than precastrated levels. The staining for the clinical specimens shows a high level of SREBP-1 compared with non-cancerous prostate tissue and SREBP-1 is decreased after hormone withdrawal therapy for 6 weeks (71). SREBP-1 and its downstream effectors, FASN and ACC, are upregulated in a TCGA cohort of prostate cancer and late-stage transgenic adenocarcinoma of mouse prostate tumor (72). Furthermore, SREBP-1 expression is positively associated with the clinical Gleason grades and promotes prostate cancer cell growth, migration, and castration-resistant progression, which may be mediated by the alterations of metabolic signaling networks, including androgen receptor (AR), lipogenesis, and NAPDH oxidase 5-induced oxidative stress (13). More importantly, the results from overexpression or RNA interference of SREBP-1c demonstrate that SREBP-1c specifically inhibits AR transactivation by binding to the endogenous AR target promoter, which may be associated with the recruitment of histone deacetylase 1, suggesting that SREBP-1 plays an important role in regulating AR-dependent prostate cancer growth (73). On the other hand, AR and mTOR bind to the regulatory region of SREBP-1 to promote its cleavage and translocation to the nucleus. The synthetic androgen R1881 can induce SREBP-1 recruitment to regulatory regions of its targets, which is abrogated by the inhibition of the mTOR signaling pathway. These data suggest that the AR/mTOR complex can promote SREBP-1 expression and activity to activate its downstreams for reprogramming lipid metabolism (74). Additionally, phospholipase Cϵ, Kruppel-like factor 5 (KLF5), and protein kinase D3 can directly or indirectly interact with SREBP-1 to control lipid metabolism in the occurrence and progression of prostate cancer (75–77). Considering the key roles of miRNAs or lncRNAs, miRNA-21 transcriptionally regulates insulin receptor substrate 1/SREBP-1 signaling pathway to promote prostate cancer cell proliferation and tumorigenesis (18). Two other miRNAs, miRNA-185 and miRNA-342, can block SREBP-1/2 and its downregulated targets, FASN and hydroxy-3-methylglutaryl CoA reductase (HMGCR), to inhibit prostate cancer tumorigenicity (78). A long non-coding RNA molecule, PCA3 is inversely correlated with miRNA-132-3p to regulate the antimony-induced lipid metabolic imbalance, which is closely related to the SREBP-1 protein level, not influencing the SREBP-2 expression in mRNA and protein levels (79). Collectively, these data from cell and animal models or clinical specimens demonstrate that SREBP-1 plays a very important role in prostate cancer progression and that multiple signaling pathways, such as AR and miRNAs, can regulate SREBP-1 activation to mediate the dysfunction of lipid metabolism in prostate cancer.

## Breast cancer

As the pattern in other cancers, the mRNA and protein levels of SREBP-1 are higher in tissues of breast cancer, compared with paracancerous tissues. A higher expression of SREBP-1 is positively correlated with tumor differentiation, metastatic stage, and metastasis in lymph nodes. Moreover, *in vitro* studies reveal that SREBP-1 inhibition can inhibit the cell migration and invasion of breast cancer cells, such as MDA-MB-231 and MCF-7 (14). In aromatase inhibitor-resistant breast cancer cells, SREBP-1 can drive Keratin-80 upregulation to directly promote cytoskeletal rearrangements, cellular stiffening, and cell invasion (80). In MCF-10a breast epithelial cells with H-ras transformation and inhibition of MAP kinase, SREBP-1c and FASN are upregulated and SREBP-1a shows less change. In a panel of samples with primary human breast cancer, SREBP-1c and FASN are increased and correlated with each other (81). miRNA expression profiling shows that miRNA-18a-5p is most evidently decreased in breast cancer cells, which can target SREBP-1 to repress E-cadherin expression. This suggests that the miR-18a-5p/SREBP-1 axis plays a crucial role in the epithelial–mesenchymal transition and metastasis of advanced breast cancer (82). A hormone derived from adipose tissue, leptin can induce ATP production from fatty acid oxidation and intracellular lipid accumulation in MCF-7 cells and tumor xenograft models, which is mediated by SREBP-1 induction and autophagy in breast cancer (83). Additionally, two key molecules, glucose-regulated protein 94 (GRP94) and nuclear protein p54(nrb)/Nono, can regulate the SREBP-1 signaling pathway in *in vitro* and *in vivo* models, which have been potential targets for breast cancer treatment (84, 85). GRP94 can upregulate genes for fatty acid synthesis and degradation, including SREBP-1, LXRα, and acyl-CoA thioesterase 7, to favor the progression of breast cancer metastasis (84). As shown in immunohistological staining for human breast cancer tissues, the level of p54(nrb) is positively correlated with SREBP-1a, and its conserved Y267 residue is required for the interaction with nuclear SREBP-1a. It suggests that p54(nrb) is a key regulator for the nuclear form of SREBP-1a, which might be a potential target for treating breast cancer (85). Taken together, SREBP-1 can favor breast cancer progression, which can be regulated by multiple signal molecules, such as miRNA-18a-5p, GRP94, and p54(nrb).

## Glioblastoma

In clinic, the therapeutic efficacy of drugs on GBM might be closely related to EGFR amplification, its mutations, or PI3K hyperactivation (86). EGFR induces the SREBP-1 cleavage and nuclear translocation, which is mediated by Akt but is independent on mTORC1 (40). EGFR-activating mutation (EGFRvIII) can promote SREBP-1 cleavage and cause a higher expression of nuclear SREBP-1 to elevate LDLR expression in GBM patient samples, tumors cells, and mouse models, which is dependent on the PI3K/Akt pathway (15, 87). Moreover, EGFR signaling binds to specific sites of the enhanced miRNA-29 promoter to increase its expression in GBM cells by upregulating SCAP/SREBP-1 whereas miRNA-29 can suppress SCAP and SREBP-1 expression to inhibit lipid synthesis in GBM (88, 89). Additionally, it is found that SREBP-1 depletion can induce apoptosis and endoplasmic reticulum stress in U87 GBM cells and tumor xenograft models, which is the result of Akt/mTORC1 signaling-mediated cancer growth and survival (90). Except EGFR and Akt/mTORC1 signaling, sterol O-acyltransferase highly expressed in GBM can control the cholesterol esterification and storage as a key player *via* upregulation of SREBP-1 (91). Collectively, the oncogenic EGFR/PI3K/Akt pathway upregulates SREBP-1 activation to control lipid metabolism in GBM.

## Hepatocellular carcinoma

Lipogenesis participates in the initiation and progression of HCC, one of the leading causes of cancer-related deaths worldwide. Multiple pathways can control SREBP-1 to regulate lipogenesis in HCC, such as hepatoma-derived growth factor (HDGF), apoptosis-antagonizing transcription factors (AATF), or acyl-CoA synthetases. HDGF is found to be closely associated with HCC prognosis, which is coexpressed with SREBP-1. The changes of the first amino acid or the type of PWWP domain are crucial for HDGF-mediated SREBP-1 activation for the lipogenesis promotion in HCC (92). In human non-alcoholic steatohepatitis, HCC tissues, and various human HCC cell lines, AATF expression is higher, which can be induced by TNF-α. Promoter analysis reveals that AATF can bind SREBP-1c to regulate HCC cell proliferation, migration, colony formation, and xenograft tumor growth and metastasis (93). As an HCC-associated tumor suppressor, zinc fingers and homeoboxes 2 (ZHX2) is negatively associated with SREBP-1c in HCC cell lines and human specimens. ZHX2 can increase miRNA-24-3p at the transcription level to induce SREBP-1c degradation for the suppression of HCC progression (94). A member of the acyl-CoA synthetase (ACS) family, acyl CoA synthetase 4 (ACSL4) has been identified as an oncogene and a novel marker of alpha-fetoprotein-high subtype HCC. ACSL4 upregulates SREBP-1 and its downstream lipogenic enzymes *via* c-Myc to modulate *de novo* lipogenesis during the progression of HCC (95). A tissue microarray analysis shows that 60 of 80 HCC tissues have a positive staining and high expression of spindlin 1 (SPIN1), which are positively associated with malignancy of HCC. SPIN1 coactivates and interacts with SREBP-1c to increase the levels of intracellular triglycerides, cholesterols, and lipid droplets, which can enhance HCC growth (96). Hepatitis B virus (HBV)-encoded X protein (HBx) significantly promotes HepG2 cell proliferation and lipid accumulation through increased protein levels of C/EBPα, SREBP-1, FASN, and ACC1 (97). Histone deacetylase 8 (HDAC8) is directly upregulated by SREBP-1 where it is coexpressed in dietary obesity models of HCC. HDAC8 can inhibit cell death mediated by p53/p21 and cell-cycle arrest at the G_2_/M phase and promote β-catenin-dependent cell proliferation by the interaction with chromatin modifier EZH2 to repress the Wnt antagonist in HCC (98). The deficiency of signal transducers and activators of transcription 5 (STAT5) causes the upregulation of SREBP-1 and peroxisome proliferator-activated receptor γ signaling. The combined loss of STAT5/glucocorticoid receptor can cause approximately 60% HCC at 12 months, which is associated with enhanced TNF-α and ROS and the activation of c-Jun N-terminal kinase 1 and STAT3 (99). Additionally, neddylation by UBC12 can prolong SREBP-1 stability with decreased ubiquitination. In human specimens of HCC and the Gene Expression Omnibus (GEO) database, the SREBP-1 level is positively correlated with UBC12 and contributes to HCC aggressiveness, which can be blocked by an inhibitor of NEDD8-activating enzyme E1, MLN4924 (100). Importantly, both mTOR signaling and SREBP-1 can increase fatty acid desaturase 2 expression to activate sapienate metabolism in the HCC cells and xenograft models (101). Certainly, SREBP-1 expression in HCC tissues is significantly higher than that in adjacent tissues, especially in large tumor sizes, high histological grade, and stage of tumor node metastasis (TNM). The level of SREBP-1 is correlated with a worse 3-year overall and disease-free survival of HCC patients, which is not dependent on the prediction for the prognosis. Moreover, SREBP-1 overexpression or knockdown can regulate cell proliferation, invasion, and migration of HCC cells (42). Especially, SREBP-1c, rather than SREBP-1a, is elevated and activated in HCC (102). Collectively, multiple upstream molecules, such as HDGF, AATF, ZHX2, ACSL4, SPIN, HBx, HDAC8, and STAT5, can regulate SREBP-1 expression, activation, and stability to promote HCC proliferation, invasion, and migration for tumor growth and metastasis.

## Lung cancer

The phosphorylation of Akt-mediated phosphoenolpyruvate carboxykinase 1 (PCK1) at S90 can reduce its gluconeogenic activity and use GTP as a phosphate donor to phosphorylate Insig1/2 in the endoplasmic reticulum, which activates SREBP signaling for lipid synthesis in cancer (103). In non-small cell lung cancer (NSCLC), PCK1 can promote nuclear SREBP-1 activation by phosphorylating Insig1/2, which is associated with TNM stage and progression of NSCLC (104). The mature form of SREBP-1 is overexpressed and correlated with Akt phosphorylation at Ser473 for lipid synthesis, which may be reversed by protein arginine methyltransferase 5 (PTMI5) knockdown in lung adenocarcinoma (105). B7-H3, a glycoprotein, results in aberrant lipid metabolism in lung cancer, which is mediated by the SREBP-1/FASN signaling pathway (106). In mutant KRAS-expressing NSCLC, KRAS increases SREBP1 expression in part by MEK1/2 signaling and SREBP-1 knockdown significantly inhibits cell proliferation through regulating mitochondrial metabolism (107). In EGFR-mutant NSCLC cells, SREBP-1 is a key feature of the resistance to gefitinib, which might be a promising target for the treatment of EGFR mutation in lung cancer (108). As that in other cancers, miRNA-29 reduces lung cancer proliferation and migration through inhibition of SREBP-1 expression by interacting with the 3′-UTR of SREBP-1 (109). Taken together, Akt activation and its mediated PCK1, B7-H3, and KRAS can regulate SREBP-1 expression and activation in lung cancer progression.

## Skin cancer

In melanomas, SREBP-1 predominantly binds to the transcription start sites of genes of *de novo* fatty acid biosynthesis (DNFA). The inhibition of DNFA or SREBP-1 by enzyme inhibitors or antisense oligonucleotides exerts obvious anticancer effects on both BRAFi-sensitive and -resistant melanoma cells (27). BRAF inhibition can induce SREBP-1 downregulation to inhibit lipogenesis, and SREBP-1 inhibition can overcome the acquired resistance to BRAF-targeted therapy in both *in vitro* and *in vivo* models (110). In addition, the PI3K/Akt/mTORC1 pathway, not by BRAF mutation, can regulate SREBP-1 activity and subsequent cholesterogenesis in melanoma (111). In another type of skin cancer, squamous cell carcinomas (SCCs), SREBP-1 can link tumor protein 63 (TP63)/KLF5 to regulate fatty acid metabolism, including fatty acid, sphingolipid, and glycerophospholipid biosynthesis, by binding to hundreds of cis-regulatory elements, which is associated with SCC viability, migration, and poor survival of patients (112). Thus, multiple signal pathways, such as BRAF, PI3K, and TP63/KLF5, can control SREBP-1 for the regulation of skin cancer progression.

## Other cancers

In esophageal tumors, SREBP1 is overexpressed and associated with worse overall survival rate of patients. Overexpressing SREBP1 in OE33 cells leads to increased biological phenotypes, including colony formation, migration, and invasion, which is mediated by reduced mesenchymal markers (vimentin, zinc finger E-box binding homeobox 1 (ZEB1), N-cadherin) and increased epithelial markers (E-cadherin). Mechanistically, SREBP-1 can regulate T-cell factor 1/lymphoid enhancer factor 1 and their target proteins, such as CD_44_ and cyclin D1, and increase the levels of SCD-1, phosphorylated GSK-3β, and nuclear β-catenin for the proliferation and metastasis of esophageal carcinoma (113, 114). SREBP-1 and ZEB1 are potential targets of miRNA-142-5p, a tumor suppressor, and they are associated with tumor progression and poor prognosis (114). Moreover, the levels of PCK1 pS90, Insig1 pS207/Insig2 pS151, and SREBP-1 are associated with the tumor, metastasis stage, and progression of esophageal squamous cell carcinoma, suggesting PCK1 activity-regulated SREBP-1 as a potential target for the diagnosis and treatment of esophageal cancer (115). In gastric cancer tissues and cells, SREBP-1c is activated and promotes the expressions of FASN and SCD-1 to mediate malignant phenotypes, which has been identified as a potential target for the treatment of gastric cancer (116). In the poorest cancer of the digestive system, pancreatic cancer, the lipogenic liver X receptor (LXR)–SREBP-1 axis controls polynucleotide kinase/phosphatase (PNKP) transcription for regulating cancer cell DNA repair and apoptosis. Compared with the adjacent tissues, the levels of LXRs and SREBP-1 are significantly reduced in the tumor tissues, which are inhibited by a small molecule, triptonide, by activating p53 and DNA strand break for cell death (117). Insulin induces transgelin-2 *via* SREBP-1-mediated transcription, which has been a novel therapeutic target for diabetes-associated pancreatic ductal adenocarcinoma (118). High glucose can enhance the SREBP-1 level to promote tumor proliferation and suppress apoptosis and autophagy (119). A high expression of TNF-α is associated with significant reductions of SREBP-1, FASN, and ACC at the mRNA level in pancreatic ductal adenocarcinoma (120). Importantly, SREBP-1 is overexpressed in both pancreatic cancer tissues and cell lines, which is critical for cancer proliferation, apoptosis, tumor growth, and overall survival (41). Therefore, the SREBP-1 signaling axis has been a promising target for the prevention and treatment of pancreatic cancer.

**Figure 2:**
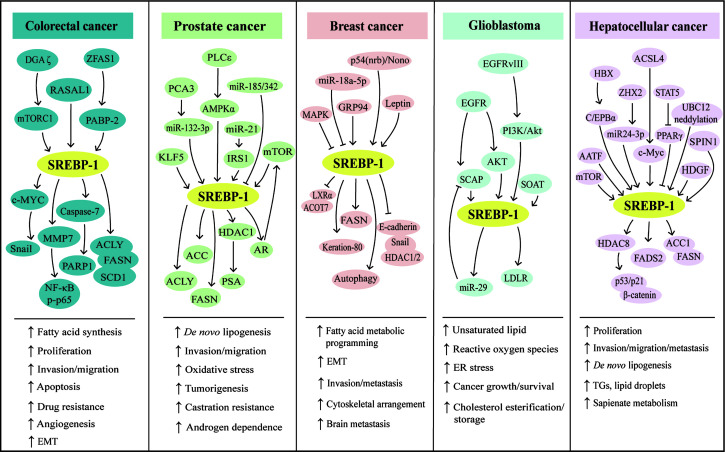
SREBP-1-mediated lipogenesis in the five types of cancers. Multiple pathways can regulate SREBP-1 and its downstream targets to mediate aggressive characteristics, including proliferation, invasion, migration, EMT, tumorigenesis, metastasis, angiogenesis, and drug resistance in colorectal, prostate, breast, hepatocellular cancer, and glioblastoma. Meanwhile, in these cancers, SREBP-1 activation can increase *de novo* lipogenesis and regulate fatty acid metabolic programming, lipid droplets, and sapienate metabolism. EMT: epithelial–mesenchymal transition, ER: endoplasmic reticulum, TGs: triglycerides.

Compared with controls, SREBP-1 is significantly increased and positively correlated with serum triglyceride in endometrial cancer (121). Ten single-nucleotide polymorphisms (SNPs) of SREBP-1 are associated with endometrial cancer, and rs2297508 SNP with the C allele accounts for 14%, which may serve as a genetic marker for early detection (122). In a progestin-resistant Ishikawa cell line, sirtuin 1 (SIRT1) and SREBP-1 at the mRNA and protein levels are upregulated, while progesterone receptor and forkhead transcription factor 1 (FoxO1) are downregulated, suggesting that the SIRT1/FoxO1/SREBP-1 pathway is involved in the progestin resistance of endometrial cancer (123). It is also found that FoxO1 can inhibit proliferative and invasive capacities by directly inhibiting SREBP-1 in this cancer (124). Salt-inducible kinase 2 can upregulate SREBP-1c to enhance fatty acid synthesis and SREBP-2 to promote cholesterol synthesis in *in vitro* and *in vivo* ovarian cancer models (125). In the obesity host, increased SREBP-1 is linked to ovarian cancer progression and metastasis (126) and meditates malignant characteristics, such as cell proliferation, migration, invasion, and tumor growth (127). In clear cell renal cell carcinoma (ccRCC) patients, high levels of E2F transcription factor 1 (E2F1) and SREBP-1 are associated with poor prognosis. E2F1 can increase ccRCC cell proliferation and epithelial–mesenchymal transition (EMT) and promote tumor progression of a mouse xenograft model by inhibiting SREBP-1-mediated aberrant lipid metabolism (128). In bladder cancer, PKM2 interacts with SREBP-1c through the regulation of the Akt/mTOR signaling pathway, which in turn activates FASN transcription for tumor growth (129). Collectively, SREBP-1-regulated lipid metabolism is essential for the cancer progression of the urinogenital system, including endometrial, ovarian, and bladder cancer and renal cell carcinomas. The results from cell lines and clinical tissues demonstrate that long intergenic non-protein-coding RNA 02570 can adsorb miRNA-4649-3p to upregulate SREBP-1 and FASN, which promotes the progression of nasopharyngeal carcinoma (NPC) (130). Epstein–Barr virus-encoded latent membrane protein 1 is expressed in NPC and increases the maturation and activation of SREBP-1 to induce *de novo* lipid synthesis in the tumor growth and metastasis, which can be inhibited by two inhibitors, luteolin and fatostatin (131). In oral squamous cell carcinoma, glutathione peroxidase 4 (GPX4) can reduce the ferroptosis-mediated cell number *via* downregulation of SREBP-1 (132). Compared with normal thyroid tissue or benign thyroid nodules, SREBP1 expression is significantly higher in invasive thyroid cancer, which is associated with extrathyroidal extension, advanced stage, and shorter survival of patients (28, 133). The *in vitro* results demonstrate that SREBP-1 can obviously increase the rate of oxygen consumption and the capacities of invasion and migration in thyroid cancer cells, which is mediated by the upregulation of the Hippo-YAP/CYR61/CTGF pathway (133). In addition, the nuclear form of SREBP-1 (nSREBP-1) can bind to the promoter of protein kinase RNA-like endoplasmic reticulum kinase (PERK) to augment its expression and phosphorylation, which might be the main reason that nSREBP-1 regulates cell proliferation, cell cycle arrest, apoptosis, and autophagy under endoplasmic reticulum (ER) stress in sarcomas (chondrosarcoma and osteosarcoma) (134). Together, SREBP-1 is upregulated and participates in the progression of NPC, oral squamous cell carcinoma, thyroid cancer, and sarcomas. Collectively, these findings indicate that the various key pathways that control SREBP-1 activation and SREBP-1-regulated lipogenesis significantly participate in tumor growth and progression and may be a promising target in multiple malignancies (**Table 2**, **Figure 2**).

**Table 2:** SREBP-1-regulated lipogenesis in different cancers.

Cancer type	Targets	Molecular mechanism	Refs
Colorectal cancer	SREBP-1/FASN	Is higher than that in non-cancerous tissues and regulates the binding on FASN promoters for cancer proliferation, invasion, and migration	(60, 62)
Colorectal cancer	SREBP-1/MMP-7/NF-κB p65 phosphorylation	Promotes angiogenesis, invasion, and metastasis	(61)
Colorectal cancer	SREBP-1/ACLY	Enhances biological behaviors, including cell proliferation, DNA reproductions, apoptosis, invasion, and migration and regulates lipid production and related metabolic pathways	(63)
Colorectal cancer	SREBP-1-mediated the alternation of cellular metabolism	Decreases fatty acid levels to inhibit the initiation and tumor progression	(64)
Colorectal cancer	SREBP-1/c-MYC/SNAIL	Enhances the binding to the SNAIL promoter to increase its expression and promote epithelial–mesenchymal transition	(65)
Colorectal cancer	SREBP-1/caspase-7	Regulates the chemosensitivity to gemcitabine or the resistance of 5-FU	(60, 66)
Colorectal cancer	SREBP-1/FASN	Is increased by radiation stimuli and causes cholesterol accumulation, cell proliferation, and apoptosis	(67)
Colorectal cancer	RAS protein activator like 1/SREBP-1c/LXRα/SCD-1	Inhibits cell proliferation and tumor growth by directly binding SREBP-1c, LXRα, and SCD-1 promoter	(68)
Colorectal cancer	LncRNA ZFAS1/PABP-2/SREBP-1	Binds polyadenylate-binding protein 2 (PABP-2) to stabilize SREBP-1 at the mRNA level to regulate lipid accumulation for malignant phenotype	(69)
Colorectal cancer	Diacylglycerol kinase zeta/mTORC1/SREBP-1	Promotes mTORC1 activation to regulate diacylglycerol and phosphatidic acid metabolism for maintaining tumor cell growth and survival	(70)
Prostate cancer	SREBP-1a/SREBP-1c	Are significantly greater than non-cancerous tissues and precastration and is decreased after hormone withdrawal therapy	(71)
Prostate cancer	SREBP-1/FASN/ACC	Are upregulated and associated with clinical Gleason grades, and castration-resistant progression	(13, 72)
Prostate cancer	SREBP-1/AR	Promotes cell growth, migration, and progression by regulating metabolic and signaling networks, including AR, lipogenesis, and oxidative stress	(13)
Prostate cancer	SREBP-1c/AR/HDAC1	Inhibits AR transactivation and is associated with the recruitment of histone deacetylase 1 for AR-dependent cancer growth	(73)
Prostate cancer	AR/mTOR/SREBP-1	Binds the regulatory region of SREBP-1 to promote the cleavage and nuclear translocation for lipid metabolism reprogramming	(74)
Prostate cancer	PLCϵ/AMPKα/SREBP-1	Is elevated and induce SREBP-1 nuclear translocation through the inhibition of AMPKα phosphorylation for cancer lipid metabolism and malignant progression	(75)
Prostate cancer	KLF5/SREBP-1/FASN	Binds to SREBP-1 to enhance the promoter activity of FASN for androgen-dependent induction	(76)
Prostate cancer	Protein kinase D3/SREBP-1/FASN/ACLY	Interacts with SREBP-1 to regulate the levels of lipogenic genes, such as FASN and ACLY for *de novo* lipogenesis and cancer progression	(77)
Prostate cancer	miRNA-21/IRS1/SREBP-1/FASN/ACC	Downregulates insulin receptor substrate 1 (IRS1)-medicated transcription to decrease the levels of SREBP-1, FASN, and ACC for tumorigenesis	(18)
Prostate cancer	miRNA-185/-342/SREBP-1/2	Blocks SREBP-1/2 and its downstream targets to inhibit tumorigenicity	(78)
Prostate cancer	lncRNA PCA3/miRNA-132-3p/SREBP-1	Is inversely correlated with the microRNA-132-3p level to interact SREBP-1 for antimony-induced lipid metabolic disorder	(79)
Breast cancer	SREBP-1	Is higher than paracancerous tissues and positively correlated with tumor differentiation, metastatic stage, and lymph node metastasis	(14)
Breast cancer	SREBP-1/keration-80	Drives keration-80 upregulation to promote cytoskeletal arrangement and invasive characteristic for aromatase inhibitor resistance	(80)
Breast cancer	MAPK/PI3K/SREBP-1c/FASN	Are upregulated and correlated with each other, and participate in the H-ras transformation and MAP kinase inhibition	(81)
Breast cancer	miRNA-18a-5p/SREBP-1/Snail/HDAC1/2	Modulates epithelial–mesenchymal transition and metastasis by regulating a complex formation of Snail and HDAC1/2	(82)
Breast cancer	SREBP-1/autophagy	Contributes to leptin-mediated fatty acid metabolic reprogramming	(83)
Breast cancer	GRP94/SREBP-1/LXRα/ACOT7	Regulates the protein levels of genes encoding fatty acid synthesis and degradation, such as SREBP-1, LXRα and ACOT7 for brain metastasis	(84)
Breast cancer	Nuclear protein p54(nrb)/Nono/SREBP-1	Is positively corelated with SREBP-1, and conserved Y267 residue is required for nuclear SREBP-1a binding and protein stability for increased lipid demand of cancer growth	(85)
Glioblastoma	EGFR/SREBP-1/Akt	Induces SREBP-1 cleavage and nuclear translocation by Akt activation, which is independent in mTORC1 for fatty acid synthesis and rapamycin’s poor efficacy	(40)
Glioblastoma	EGFR activating mutation/SREBP-1/LDLR	Promotes the cleavage of SREBP-1 to increase the expression of the nuclear form for increased LDLR by activating the PI3K/Akt pathway	(15)
Glioblastoma	EGFR/SCAP/SREBP-1/miRNA-29	Binds specific sites of microRNA-29 for its increased expression by upregulating SCAP/SREBP-1	(88)
Glioblastoma	miRNA-29/SCAP/SREBP-1	Represses the levels of SCAP and SREBP-1 for the inhibition of *de novo* lipid metabolism	(89)
Glioblastoma	SREBP-1/Akt/mTORC1	Regulates endoplasmic reticulum stress and apoptosis by Akt/mTORC1 signaling	(90)
Glioblastoma	Sterol O-acyltransferase/SREBP-1	Controls cholesterol esterification and storage by upregulating SREBP-1	(91)
Hepatocellular carcinoma	Hepatoma-derived growth factor/SREBP-1	Is coexpressed with and activates SREBP-1 by changing first amino acid or the type of PWWP domain for cancer development and prognosis	(92)
Hepatocellular carcinoma	Apoptosis-antagonizing transcription factor/SREBP-1	Interacts with the SREBP-1c binding site to regulate cell proliferation and survival for driving hepatocarcinogenesis	(93)
Hepatocellular carcinoma	Zinc fingers and homeoboxes 2/miRNA-24-3p/SREBP-1c	Increases the microRNA-24-3p mRNA level to induce SREBP-1c degradation for suppressing cancer progression	(94)
Hepatocellular carcinoma	Acyl CoA synthetase 4/c-Myc/SREBP-1	Upregulates SREBP-1 and its downstreams by c-Myc for the regulation of *de novo* lipogenesis	(95)
Hepatocellular carcinoma	Spindlin 1/SREBP-1c	Coactivates and interacts SREBP-1c to increase the contents of intracellular triglycerides, cholesterols, and lipid droplets for cancer growth	(96)
Hepatocellular carcinoma	Hepatitis B virus-encoded X protein/C/EBPα/SREBP-1/FASN/ACC1	Promotes cell proliferation and lipid accumulation by increasing C/EBPα, SREBP-1, FASN, and ACC1	(97)
Hepatocellular carcinoma	SREBP-1/HDAC8	Directly upregulates histone deacetylase 8 and are coexpressed in tumor models induced dietary obesity	(98)
Hepatocellular carcinoma	Signal transducers and activators of transcription 5 (STAT5)/PPARγ/JNK1/STAT3	Regulates SREBP-1 and PPARγ signaling in 60% HCC by enhancing TNF-α, ROS, c-Jun N-terminal kinase 1, and STAT3	(99)
Hepatocellular carcinoma	UBC12 neddylation/SREBP-1	Prolong SREBP-1 stability with decreased ubiquitination for the contribution of cancer aggressiveness	(100)
Hepatocellular carcinoma	mTOR/SREBP-1/FADS2	Increase fatty acid desaturase 2 expression to upregulate sapienate metabolism in the cell and xenograft models	(101)
Hepatocellular carcinoma	SREBP-1c	Is higher in large tumor size, high histological grade, and stage of tumor-node metastasis, correlates with overall and disease-free survival, and regulates cell proliferation, migration, and invasion	(42, 102)
Lung cancer	Phosphoenolpyruvate carboxykinase 1/Insig1/2/SREBP-1	Phosphorylates Insig1/2 to activate SREBP-1 signaling for lipid biosynthesis, which contributes to the stage of tumor-node metastasis and progression	(103, 104)
Lung cancer	Protein arginine methyltransferase 5/SREBP-1/Akt	Catalyzes the symmetric dimethyl arginine modification of mature form of SREBP-1 to induce its activation, which is correlated with Akt phosphorylation at Ser473 for *de novo* lipogenesis and cancer growth	(105)
Lung cancer	B7-H3/SREBP-1/FASN	Modulates the SREBP-1/FASN pathway to mediate abnormal lipid metabolism	(106)
Lung cancer	KRAS/MEK1/2/SREBP-1	Increases SREBP-1 expression partly by regulating MEK1/2 signaling for cell proliferation and mitochondrial metabolism	(107)
Lung cancer	SREBP-1	Is an essential characteristic for acquired gefitinib resistance in EGFR-mutant cancer	(108)
Lung cancer	miRNA-29	Interacts with the 3′-UTR of SREBP-1 to reduce SREBP-1 expression for the inhibitions of cell proliferation, migration, and tumor growth	(109)
Melanomas	SREBP-1/ACLY/FASN/SCD	Binds to the transcription start sites of genes encoding *de novo* fatty acid biosynthesis in both BRAFi-sensitive and -resistant cells	(27)
Melanomas	SREBP-1	Mediates the acquired resistance to BRAF-targeted therapy in the *in vitro* and *in vivo* models	(110)
Melanomas	PI3K/Akt/mTORC1/SREBP-1	Regulates SREBP-1 activity and subsequent cholesterogenesis for reactive oxygen species-induced damage and lipid peroxidation	(111)
Squamous cell carcinomas	SREBP-1/tumor protein 63/KLF5	Links tumor protein 63/KLF5 to regulate fatty acid metabolism by binding hundreds of cis-regulatory elements for cancer cell viability, migration, and poor survival of patients	(112)
Esophageal cancer	SREBP-1/SCD/Wnt/β-catenin	Is overexpressed and correlated with worse overall survival of patients and regulates cell proliferation and metastasis by inducing epithelial–mesenchymal transition *via* SCD1-induced activation of the Wnt/β-catenin pathway	(113)
Esophageal cancer	SREBP-1/miR-142-5p/ZEB1	Are targeted by miR-142-5p for the migration and sphere formation *via* the reduction of epithelial–mesenchymal transition markers	(114)
Esophageal cancer	Akt/PCK1/Insig1/2 and nuclear SREBP-1	Are higher in cancer specimens and positively correlated with tumor, node and metastasis stage and progression, and poor prognosis	(115)
Gastric cancer	SREBP-1c/FASN/SCD-1	Is activated and promotes the expression of genes encoding fatty acid synthesis, such as SCD-1 and FASN for malignant phenotypes	(116)
Pancreatic cancer	Liver X receptors/SREBP-1	Are significantly reduced in tumor tissues and control the transcription of polynucleotide kinase/phosphatase for regulating DNA repair and cell death	(117)
Pancreatic cancer	Transgelin-2/SREBP-1	Is expressed in cancer tissues and indicates poor survival of patients, which induces SREBP-1-mediated transcription under the stimulation of insulin	(118)
Pancreatic cancer	High glucose microenvironment/SREBP-1	Is associated with poor prognosis and regulates cancer proliferation, apoptosis, and autophagy by enhancing SREBP-1 expression	(119)
Pancreatic cancer	TNF-α/SREBP-1/FASN/ACC	Reduces cell migration and is associated with reduced SREBP-1, FASN, and ACC involved in lipogenesis, inflammation, and metastasis	(120)
Pancreatic cancer	SREBP-1/FASN/ACC/SCD-1	Is significantly higher in cancer tissues and predicts poor prognosis and regulates lipid metabolism and tumor growth by regulating its downstream targets, FASN, ACC, SCD-1	(41)
Endometrial cancer	SREBP-1	Is significantly increased in cancer tissues and positively correlates with serum triglyceride	(121)
Endometrial cancer	SREBP-1	Ten single-nucleotide polymorphisms are associated with increased risk and rs2297508 SNP with the C allele serves as a genetic factor for early detection	(122)
Endometrial cancer	SIRT1/FoxO1/SREBP-1	Are changed in progestin-resistant cells and are involved in the development of progestin resistance	(123)
Endometrial cancer	FoxO1/SREBP-1	Inhibits cell migration and invasion abilities and tumorigenesis *in vitro* and *in vivo* by directly targeting SREBP-1	(124)
Ovarian cancer	Salt-inducible kinase-2/SREBP-1c/FASN	Promotes fatty acid synthesis by upregulating SREBP-1c expression and FASN, which is mediated by the PI3K/Akt pathway	(125)
Ovarian cancer	SREBP-1	Is higher in cancer tissues compared to benign and borderline tumors and regulates cell growth, invasion, migration, and apoptosis in the cell and mouse xenograft models	(126, 127)
Clear cell renal cell carcinoma	E2F transcription factor 1/SREBP-1	Is associated with poor prognosis and increases cell proliferation, epithelial–mesenchymal transition, and tumor progression by activating SREBP-1-mediated fatty acid biosynthesis	(128)
Bladder cancer	Pyruvate kinase 2/Akt/mTOR/SREBP-1c/FASN	Physically interacts with SREBP-1c to regulate FASN transcription for tumor growth by regulating Akt/mTOR signaling	(129)
Nasopharyngeal carcinoma	Long intergenic non-protein coding RNA 02570/miRNA-4649-3p/SREBP-1/FASN	Is upregulated in the late clinical stage and adsorbs microRNA-4649-3p to upregulate SREBP-1 and FASN for cancer progression	(130)
Nasopharyngeal carcinoma	Epstein–Barr virus-encoded latent membrane protein 1/SREBP-1	Is overexpressed in cancer tissues and increases the expression, maturation, and activation of SREBP-1 for inducing *de novo* lipid synthesis and is involved in cancer pathogenesis driven by Epstein–Barr virus E	(131)
Oral squamous cell carcinoma	Glutathione peroxidase 4/SREBP-1/ferroptosis	Is higher in cancer cells, regulates cell proliferation and ferroptosis, and is correlated with p53 immunoreactivity	(132)
Thyroid cancer	SREBP-1	Is significantly higher in invasive cancer tissues and associated with advanced disease stage and short survival	(28)
Thyroid cancer	SREBP-1/Hippo-YAP/CYR61/CTGF	Obviously increases oxygen consumption rate, invasion, and migration by upregulating the Hippo-YAP/CYR61/CTGF pathway	(133)
Sarcomas	Nuclear form of SREBP-1/PKR/PERK	Binds the promoter of protein kinase RNA(PKR)-like endoplasmic reticular kinase (PERK) to augment its expression and phosphorylation for malignant phenotypes	(134)

GRP94, glucose-regulated protein 94; ACTO7, acyl-CoA thioesterase 7; SOAT, sterol O-acyltransferase.

## Targeting the SREBP-1 signaling pathway for cancer therapy

As reported, SREBP-1 has been a potential target and its inhibition by small molecules or natural products has been potential therapeutics for preventing and treating cancer (12). A specific inhibitor of SREBP activation, fatostatin is a diarylthiazole derivative and binds to SCAP to inhibit the translocations of SREBP-1 and SREBP-2 from the ER to Golgi (135, 136). In prostate cancer, fatostatin suppresses cell proliferation and colony formation in both androgen-responsive or -insensitive cancer cells and causes G_2_/M cell cycle arrest and cell death, which is mediated by the blockade of the SREBP-regulated metabolic pathway and AR signaling network (136). Fatostatin can inhibit SREBP activity to influence the assembly of mitotic microtubule spindle and cell division in various cancer cells (137). In breast cancer, fatostatin is more sensitive to estrogen receptor-positive cells in response with cell cycle arrest and apoptosis through accumulation of lipids under ER stress, such as polyunsaturated fatty acids (PUFAs), not the inhibition of SREBP activity (138). Moreover, fatostatin can reverse progesterone resistance through inhibition of the SREBP-1/NF-κB pathway in endometrial cancer (139). In pancreatic cancer, both fatostatin and PF429242 inhibit cell proliferation and the growth of xenograft tumor by reducing SREBP-1 and its downstream signaling cascades, such as FASN and SCD-1 (17). Additionally, the combination of fatostatin with tamoxifen has a synergistic effect on the inhibition of PI3K/Akt/mTOR signaling in ER-positive breast cancer (140). Together, fatostatin mainly regulates the activation of SREBP-1/2 to block different cancer progressions, while it also regulates the accumulations of PUFAs and the PI3K/Akt/mTOR pathway. Nelfinavir, a HIV protease inhibitor, induces ER stress and caspase-dependent apoptosis and increases SREBP-1 protein half-life by blocking intracellular trafficking of SREBP-1 and activating transcription factor 6 (ATF6) from site-2 protease (S2P) inhibition in liposarcoma cells and xenograft mouse models (141). In prostate cancer, nelfinavir inhibits AR activation and nuclear translocation of SREBP-1 and ATF6 by regulating S2P-mediated intramembrane proteolysis, which is associated with ER stress, inhibition of unfolded protein response, apoptosis, and autophagy for treating castration-resistant prostate cancer (142). In addition, nelfinavir and its analogs, #6, #7, and #8, have more potent effects on S2P cleavage than 1,10-phenanthroline, a metalloprotease-specific inhibitor. These molecules can block S2P cleavage activity to lead to the accumulations of precursor SREBP-1 and ATF6 against castration-resistant prostate cancer (143). These findings indicate that nelfinavir is a potential agent targeting S2P cleavage for cancer therapy. Currently, several targeted drugs targeting tyrosine kinase can regulate the SREBP-1 pathway to play anticancer effects. Osimertinib, the first-approved third-generation EGFR inhibitor, facilitates SREBP1 degradation and reduces the levels of its targets and lipogenesis in EGFR-mutant NSCLC cells and tumors, which suggests an effective strategy for overcoming acquired resistance of EGFR inhibitors by targeting SREBP-1 (144). Sorafenib, a multikinase inhibitor targeting RAS/MEK/ERK, VEGFR, and PDGFR, significantly affects SCD-1 expression to decrease the synthesis of monounsaturated fatty acids and suppresses ATP production to activate AMPK, which can reduce the levels of SREBP-1 and phosphorylate mTOR for the inhibition of liver cancer (145).

Furthermore, several small molecules or new dosage forms are reported as the regulators for SREBP-1-regulated lipogenesis. An AR degradation enhancer, ASC-J9 suppresses PCa cell growth and invasion by the AR/SREBP-1/FASN pathway in AR-positive cells and PI3K/Akt/SREBP-1/FASN signaling in AR-negative cells, which indicates that it mainly suppresses FASN-mediated PCa progression in both AR-dependent/independent manners (146). A newly developed AR antagonist, proxalutamide, significantly inhibits proliferation and migration, induces the caspase-dependent apoptosis, and diminishes the level of lipid droplets in PCa cells by regulating the levels of ACL, ACC, FASN, and SREBP-1. Moreover, proxalutamide can decrease AR expression in PCa cells, which may overcome the resistance of AR-targeted therapy (147). Leelamine, a pyruvate dehydrogenase kinase inhibitor, can downregulate the expressions of SREBP-1 and key fatty acid synthesis enzymes (ACLY, ACC1, FASN) at the mRNA and protein levels to suppress fatty acid synthesis against PCa progression (148). An HDAC inhibitor, valproic acid can inhibit prostate cancer cell viability and induce apoptosis by regulating the C/EBPα/SREBP-1 pathway based on the results of *in vitro* and *in vivo* experiments (149). A novel anti-beta2-microglobulin monoclonal antibody decreases cell proliferation, induces massive cell death, and decreases AR expression *via* inhibition of SREBP-1-mediated fatty acid synthesis in the models of multiple PCa cell lines and xenograft tumor (150). N-Arachidonoyl dopamine, a typical representative of N-acyl dopamines, can inhibit breast cancer cell migration, EMT, and stemness and cause decreased cholesterol biosynthesis by inhibiting SREBP-1, its key targets, and endoplasmic reticulum kinase 1/2 (ERK1/2) pathways (151). A novel small-molecule, SI-1, 1-(4-bromophenyl)-3-(pyridin-3-yl) urea inhibits aerobic glycolysis and enhances the antitumor effect of radiofrequency ablation in the HCC cells and xenograft tumors *via* inhibition of SREBP-1 activation (152). The treatment of docosahexaenoic acid, but neither n-6 PUFA arachidonic acid nor oleic acid, can inhibit the levels of the precursor of SREBP-1 and its mature form, and FASN is induced by estradiol and insulin to mediate breast cancer proliferation, which is the result from reduced phosphorylated Akt, not from ERK1/2 phosphorylation (153). In different GBM cells, phytol and retinol show cytotoxic effects at dose dependence, which might be mediated by the levels of SREBP-1, FASN, and farnesyl-diphosphate farnesyltransferase (FDFT1) to downregulate cholesterol and/or fatty acid biosynthetic pathways (154). In addition, it is reported that platinum complexes or micelles can regulate the SREBP-1 pathway to play a more effective function against cancer growth and progression. The pyridine co-ligand-functionalized cationic complexes, including C2, C6, and C8, can suppress cancer invasion, migration, and tumor spheroid formation through inhibition of SREBP-1-mediated lipid biogenesis (155). PB@LC/D/siR is synthesized by cross-linking for docetaxel and siSREBP1 delivery fused with PCa cell membranes and bone marrow mesenchymal stem cells, which show the enhanced antitumor effects in bone metastatic castration-resistant PCa, with the characteristics of deep tumor penetration, high safety, and bone protection *via* downregulation of SREBP-1 and SCD-1 at the mRNA level (156).

Importantly, natural products can regulate SREBP-1-regulated lipogenesis for the prevention and treatment of different cancers, including CRC, PCa, HCC, and breast cancer. In CRC, there are four compounds for regulating multiple signaling pathway-medicated SREBP-1 activation. One of ginger derivatives, 6-shogaol, can attenuate the adipocyte-conditioned medium effect by controlling the SREBP-1 level mediated by Akt, p70S6K, and AMPK signaling pathways in 5-FU-treated CRC (157). Ilexgenin A, the main bioactive compound from *Ilex hainanensis Merr*., significantly inhibits inflammatory colitis of mice induced by azoxymethane/dextran sulfate sodium and reverses the metabolites associated with colorectal cancer, which might be mediated by reprogramed lipid metabolism of the HIF-1α/SREBP-1 pathway (158). RA-XII, a natural cyclopeptide isolated from *Rubia yunnanensis*, decreases the motility of HCT 116 cells *via* inhibition of the β-catenin pathway and inhibits CRC growth and metastasis by downregulating the levels of SREBP-1, FASN, and SCD for restraining lipogenesis (159). Berberine inhibits colon cancer cell proliferation and induces cell cycle arrest of the G_0_/G_1_ phase *via* the Wnt/β-catenin pathway. Importantly, berberine blocks SREBP-1 activation and SCAP expression to downregulate the levels of lipogenic enzymes against tumor growth (160). The studies report that three compounds have effective anti-HCC effects by inhibiting SREBP-1 and its downstream targets. Cinobufotalin is extracted from the skin secretion of the giant toad and can effectively promote cell apoptosis, induce cell cycle G_2_/M arrest, and inhibit cell proliferation by downregulating SREBP-1 expression and the interaction with sterol regulatory elements for the inhibition of *de novo* lipid synthesis in HCC (161). Moreover, emodin, a main active component from *Reynoutria multiflora* (*Thunb.*) *Moldenke*, triggers apoptosis and reduces mitochondrial membrane potential by regulating intrinsic apoptosis signaling pathway in HCC cells. SREBP-1 and its downstream targets, such as ACLY, ACCα, FASN, and SCD-1, are inhibited to mediate the anticancer effect of emodin against HCC (162). Additionally, In HCC cells and xenograft tumors, betulin treatment inhibits cellular glucose metabolism to prevent metastatic potential and also facilitates the inhibitory effect of sorafenib on HCC through inhibition of SREBP-1 (163). In breast cancer cells, theanaphthoquinone, a member of the thearubigins generated by the oxidation of theaflavin, can block the EGF-induced nuclear translocation of SREBP-1 and also modulate the phosphorylation of ERK1/2, Akt, and EGFR/ErbB-2 induced by EGF, which can cause the blockade of FASN for the inhibition of cell viability and induction of cell death (164). Furthermore, in HER2-overexpressing breast cancer cells, piperineis strongly inhibits proliferation, induces apoptosis, and enhances paclitaxel sensitization through inhibition of SREBP-1/FASN signaling, which might be mediated by HER2 expression, ERK1/2, p38, and Akt signaling pathways (165). Additionally, vitexin and syringic acid from foxtail millet bran can inhibit breast cancer proliferation and block the conversion of saturated fatty acids to monounsaturated fatty acids, which is mediated by the decreases of glucose regulated protein 78 and SREBP-1, and its target, SCD-1 (166).

In NSCLC, ginsenoside Rh2 suppresses SREBP-1 expression and its nuclear translocation to disturb the interaction of SREBP-1 and FASN, which can enhance the immune effect and have a synergistic antitumor effect with cyclophosphamide (167). In gallbladder cancer, α-mangostin, a dietary xanthone, represses the proliferation, clone formation ability, and *de novo* lipogenesis; induces cell cycle arrest and apoptosis; and enhances gemcitabine sensitivity of gallbladder cancer cells, which might be mediated by AMPK activation and the inhibition of nuclear SREBP-1 translocation in cancer cells and tumor xenograft models (168). In cervical carcinoma, quercetin, a naturally occurring dietary flavonoid, decreases cell proliferation and induces cell death in Hela cells by reducing the O-GlcNAcylation of AMPK and the interaction of OGT and SREBP-1 (169). In both *in vitro* and *in vivo* models of pancreatic cancer, resveratrol induces gemcitabine chemosensitivity and suppresses sphere formation and markers of cancer stem cells by targeting SREBP-1, which indicates that resveratrol can be an effective sensitizer for chemotherapy (170). Timosaponin A3 can inhibit SREBP-1 and its targets, FASN and ACC, to reduce cell viability and increase cell cycle arrest and apoptosis of BxPC-3 cells and pancreatic cancer xenograft growth, which is independent in the Akt/GSK-3β pathway (171).

**Figure 3:**
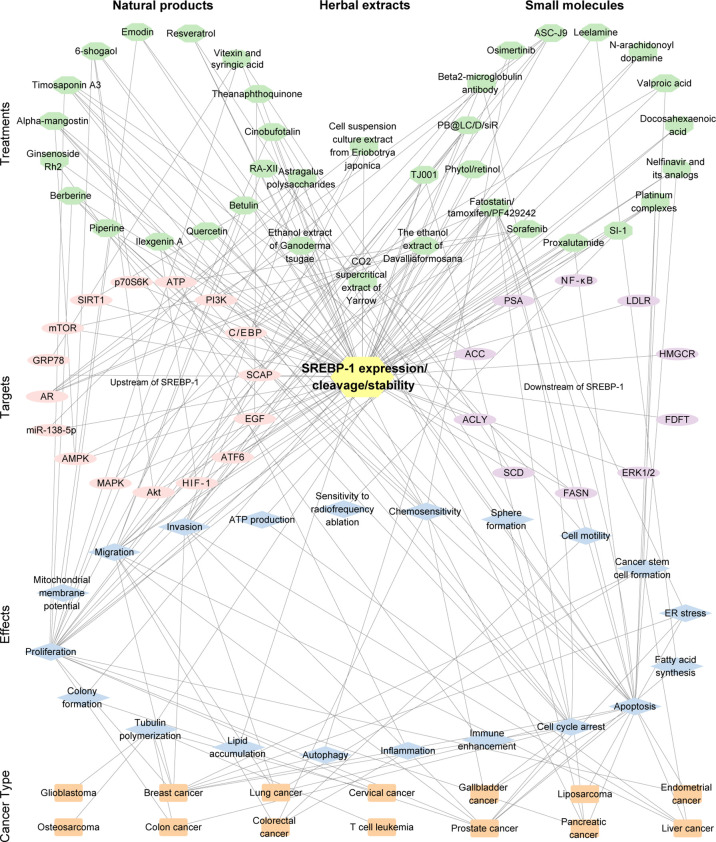
The interaction of the treatment by small molecules, natural products, or the extract of herbs, the targets (SREBP-1 expression/cleavage/stability), effects, and cancer types. The pink/purple circles represent upstream and downstream targets of SREBP-1. The blue diamonds represent the effects from the inhibition of the SREBP-1 pathway. The orange rectangles represent cancer types. The number of edges connected between the nodes in the network represents the count of their connections.

Besides these natural compounds, the extract, polysaccharides, or decoction from various herbs can also regulate the SREBP-1-mediated lipogenesis against PCa and pancreatic cancer growth and progression. In PCa, the ethanol extract of Ganoderma tsugae, a Chinese natural and herbal product, significantly inhibits the expressions of SREBP-1 and its downstream genes associated with lipogenesis and downregulates the levels of AR and PSA to block cancer growth and progression with androgen response and castration resistance (172). The results from the *in vitro* and *in vivo* experiments demonstrated that the ethanol extract of Davallia formosana can suppress proliferation, migration, and invasion in PCa cells by inhibiting the levels of SREBP-1, FASN, AR, and PSA, suggesting that SREBP-1/FASN/lipogenesis and the AR axis could be potential targets for the treatment of PCa (173). The cell suspension culture extract from *Eriobotrya japonica* significantly inhibits PCa cell growth, migration, and invasion by decreasing the SREBP-1/FASN-mediated lipid metabolism and AR signaling pathway in the cell and mouse models (174). Moreover, the polysaccharides from *Astragalus membranaceus* (APS) greatly inhibit the proliferation and invasion of PCa cells in a dose-dependent and time-dependent manner. Mechanistic studies demonstrate that APS treatment reduces the expressions of miR-138-5p, SIRT1, and SREBP-1 to block tumorigenesis and lipid metabolism in PCa (175). In addition, a traditional herbal decoction TJ001 has significant cytotoxicity, induces cell cycle arrest at the G_1_/S stage, and inhibits lipid accumulation in D145 and PCa cells with p53 mutation by regulating the ACC expression, SREBP-1 proteolytic cleavage, and the inhibition of AMPK/mTOR, which suggest that the combination of mutant p53 targeting and TJ001 can be considered as the potential strategy for PCa treatment (176). In pancreatic cancer, the CO_2_ supercritical extract of Yarrow (*Achillea millefolium*) can downregulate the levels of SREBP-1, FASN, and SCD to induce cytotoxicity in cancer cells and diminish tumor growth of xenograft mouse models, which can be developed as a complementary adjuvant or nutritional supplement (177). Taken together, the current findings of targeting SREBP-1-mediated lipogenesis by small molecules, natural products, or herb extracts are summarized in **Table 3** and **Figure 3**.

**Table 3:** Targeting SREBP-1-mediated lipogenesis in different cancers.

Treatment	Targets	Cancer type	Molecular mechanism	Refs
Fatostatin	SREBP-regulated metabolic pathway and AR signaling	Prostate cancer	Suppresses cell proliferation and colony formation and causes G2/M cell cycle arrest and cell death in both androgen-responsive or insensitive cancer cells	(136)
Fatostatin	SREBP activity	Various cancers	Possesses antitumor anti-mitotic properties by inhibiting tubulin polymerization and activating spindle assembly checkpoints	(137)
Fatostatin	Accumulation of polyunsaturated fatty acids	Breast cancer	Induces cell cycle arrest and apoptosis through the accumulation of lipids in response to ER stress, not by SREBP activity in estrogen receptor-positive cancer cells	(138)
Fatostatin	SREBP-1/NF-κB pathway	Endometrial cancer	Reverses progesterone resistance to inhibit proliferation and induces apoptosis both *in vitro* and *in vivo* models	(139)
Fatostatin and PF429242	SREBP-1 and its downstream targets, FASN, SCD-1	Pancreatic cancer	Inhibits cell viability and proliferation in a time- and dose-dependent manner	(17)
Fatostatin combined with tamoxifen	PI3K/Akt/mTOR pathway	Breast cancer	Significantly suppresses cell viability and invasion and regulates apoptosis and autophagy in the cell and mouse xenograft models	(140)
Nelfinavir	Intramembrane proteolysis of SREBP-1 and ATF6	Liposarcoma	Induces the increases of SREBP-1 and ATF6 resulted from S2P inhibition against ER stress and caspase-mediated apoptosis	(141)
Nelfinavir	Intramembrane proteolysis of SREBP-1 and ATF6	Prostate cancer	Inhibits androgen receptor activation and nuclear translocation of SREBP-1 to cause unprocessed SREBP-1 and ATF6 accumulations for the inhibition of proliferation and unfolded protein response	(142)
Nelfinavir and its analogs, #6, #7, and #8	Intramembrane proteolysis of SREBP-1 and ATF6	Prostate cancer	Induces the increases of SREBP-1 and ATF6 resulted from S2P inhibition against ER stress and apoptosis	(143)
Osimertinib	SREBP-1	Non-small cell lung cancer	Facilitates SREBP-1 degradation, reduces the levels of its targets and lipogenesis to overcome the acquired resistance of EGFR inhibitors	(144)
Sorafenib	ATP/AMPK/mTOR/ SREBP-1	Hepatocellular carcinoma	Suppresses ATP production to activate AMPK and reduce SREBP-1 and phosphorated mTOR levels to disrupt SCD-1-mediated synthesis of monounsaturated fatty acids	(145)
ASC-J9	AR/SREBP-1/FASN and PI3K/Akt/SREBP-1/FASN	Prostate cancer	Suppresses cell growth and invasion in both AR-dependent and AR-independent manner	(146)
Proxalutamide	SREBP-1/ACL/ACC/FASN	Prostate cancer	Inhibits the proliferation and migration, induces the caspase-dependent apoptosis, and diminishes the level of lipid droplets, which also overcome the resistance of AR-targeted therapy by decreasing AR expression	(147)
Leelamine	SREBP-1, ACLY, FASN, and SCD	Prostate cancer	Suppresses fatty acid synthesis in the cancer cells and tumor xenograft, not affected by AR status	(148)
Valproic acid	C/EBPα/SREBP-1 pathway (FASN, ACC1)	Prostate cancer	Inhibits cell viability and lipogenesis and induce apoptosis in the cancer cells	(149)
Beta2-microglobulin antibody	MAPK, SREBP-1 and AR	Prostate cancer	Decreases cell proliferation, induces apoptosis, and reduces AR expression to suppress tumor growth and progression	(150)
N-Arachidonoyl dopamine	SREBP-1/ERK1/2 pathways	Breast cancer	Inhibits cell migration, EMT, and stemness and causes decreased cholesterol biosynthesis	(151)
SI-1	SREBP-1 activation	Hepatocellular carcinoma	Enhances the sensitivity to radiofrequency ablation in cancer cells, xenograft tumors, and the patients	(152)
Docosahexaenoic acid	Precursor and mature SREBP-1, FASN	Breast cancer	Inhibits cancer cell proliferation induced by estradiol and insulin, which is dependent on Akt phosphorylation, not ERK1/2 phosphorylations	(153)
Phytol and retinol	SREBP-1, FASN, and farnesyl-diphosphate farnesyltransferase	Glioblastoma	Have cytotoxic effects in a dose-dependent manner and inhibit cholesterol and/or fatty acid biosynthetic pathways	(154)
Platinum complexes, C2, C6, C8	SREBP-1-regulated metabolic pathway (LDLR, FASN, HMGCR)	Breast, liver, and lung cancer	Inhibits invasion, migration, and cancer stem cell formation and induce apoptosis *in vitro*	(155)
PB@LC/D/siR	SREBP-1 and SCD-1	Bone metastatic castration-resistant PCa	Shows the enhanced antitumor effects with the characteristics of deep tumor penetration, high safety, and bone protection	(156)
6-Shogaol	Akt, p70S6K, and AMPK-mediated SREBP-1 levels	Colorectal cancer	Attenuates the effect of adipocyte-conditioned medium on 5-FU resistance	(157)
Ilexgenin A	HIF-1α/SREBP-1 pathway	Colorectal cancer	Inhibits azoxymethane/dextran sulfate sodium-induced inflammatory colitis and reverses colorectal cancer-associated metabolites by reprogramed lipid metabolism	(158)
RA-XII	SREBP-1, FASN, and SCD	Colorectal cancer	Decreases cell motility, tumor growth, and metastasis by restraining lipogenesis	(159)
Berberine	SCAP/SREBP-1 pathway	Colon cancer	Inhibits cell proliferation and induces G_0_/G_1_ cell cycle arrest and regulates the levels of lipogenic enzymes in the *in vitro* and *in vivo* studies	(160)
Cinobufotalin	SREBP-1 expression and the interaction with sterol regulatory elements	Hepatocellular carcinoma	Induces cell cycle G2-M arrest and apoptosis and inhibits cell proliferation *via* the inhibition of *de novo* lipid synthesis	(161)
Emodin	SREBP-1 and its downstream targets, ACLY, ACCα, FASN, and SCD-1	Hepatocellular carcinoma	Triggers apoptosis and reduces mitochondrial membrane potential to play an anticancer effect	(162)
Betulin	SREBP-1	Hepatocellular carcinoma	Inhibits cellular glucose metabolism to prevent metastatic potential and facilitates the inhibitory effect of sorafenib	(163)
Theanaphthoquinone	EGF-induced nuclear translocation of SREBP-1 and FASN expression	Breast cancer	Decreases cell viability and induces cell death by regulating ERK1/2 and Akt phosphorylation and EGFR/ErbB-2 pathways	(164)
Piperine	SREBP-1 and FASN expression	HER2-overexpressing breast cancer	Inhibits proliferation, induces apoptosis, and enhances sensitization to paclitaxel by regulating ERK1/2, p38 MAPK, and Akt signaling pathways	(165)
Vitexin and syringic acid	GRP78/SREBP-1/SCD-1	Breast cancer	Inhibits cell proliferation and impairs tumor growth by regulating cellular membrane and lipid droplet	(166)
Ginsenoside Rh2	SREBP-1 nuclear translocation and FASN	Non-small cell lung cancer	Enhances the immune effect and has a synergistic antitumor effect with cyclophosphamide	(167)
α-Mangostin	AMPK activation and nuclear SREBP-1 translocation	Gallbladder cancer	represses cell proliferation, clone formation ability, and *de novo* lipogenesis; induces cell cycle arrest and apoptosis; and enhances gemcitabine sensitivity	(168)
Quercetin	SREBP-1 and the interaction with O-linked N-acetylglucosamine transferase	Cervical cancer	In cervical carcinoma, quercetin, a naturally occurring dietary flavonoid, decreases cell proliferation and induces cell death in Hela cells by reducing the O-GlcNAcylation of AMPK	(169)
Resveratrol	SREBP-1	Pancreatic cancer	Induces gemcitabine chemosensitivity and suppresses sphere formation and the markers of cancer stem cells in both *in vitro* and *in vivo* models	(170)
Timosaponin A3	SREBP-1 and its downstream targets, FASN, ACC	Pancreatic cancer	Inhibits cell viability and induces cell cycle arrest, apoptosis in the cancer cells and xenograft model, which is independent in the Akt/GSK-3β pathway	(171)
Ethanol extract of *Ganoderma tsugae*	SREBP-1 and its downstream genes, AR, PSA	Prostate cancer	Inhibits cancer growth and activates apoptosis by blocking the SREBP-1/AR axis	(172)
Ethanol extract of *Davallia formosana*	SREBP-1/FASN/lipogenesis and AR axis	Prostate cancer	Suppresses cancer proliferation, migration, and invasion by inhibiting the levels of SREBP-1, FASN, AR, and PSA in *in vitro* and *in vivo* experiments	(173)
Cell suspension culture extract from *Eriobotrya japonica*	SREBP-1/FASN and AR signaling pathways	Prostate cancer	Inhibits cell growth, migration, and invasion by decreasing the SREBP-1/FASN-mediated lipid metabolism and AR signaling pathway in the cell and mouse models	(174)
Astragalus polysaccharides	miR-138-5p/SIRT1/SREBP-1 pathway	Prostate cancer	Inhibits proliferation and invasion in a dose- and time-dependent manner and blocks tumorigenesis and lipid metabolism by the miR-138-5p/SIRT1/SREBP-1 pathway	(175)
TJ001	ACC expression, SREBP-1 proteolytic cleavage,	Prostate cancer	Has significant cytotoxicity, induces cell cycle arrest at the G_1_/S stage, and inhibits lipid accumulation in D145 cells by regulating the AMPK/mTOR pathway	(176)
CO2 supercritical extract of Yarrow	SREBP-1, FASN, and SCD	Pancreatic cancer	Induces cytotoxicity in cancer cells and diminishes tumor growth of the xenograft mouse model	(177)

## Conclusions

This review mainly summarized the recent findings of SREBP-1-mediated lipogenesis in different cancer and as potential targets by small molecules, natural products, or the extracts of herbs against cancer growth and progression. Multiple signaling pathways, such as EGFR and PI3K/Akt/mTOR, control SREBP-1 expression and activation to regulate the transcription of multiple genes for fatty acid synthesis (ACLY, ACC, FASN, SCD-1/5) and lipid uptake (LDLR) in human cancer. Multiple signaling molecules can regulate SREBP-1 expression, activation, stability, and binding. Furthermore, SREBP-1 can regulate downstream signaling pathways to mediate cancer proliferation, apoptosis, endoplasmic reticulum stress, and epithelial–mesenchymal transition for tumor growth and metastasis of different cancers, including colon, prostate, breast, lung, and hepatocellular cancer. Additionally, many studies have demonstrated that fatostatin, nelfinavir, AR antagonist, natural compounds, or herbal extracts can target SREBP-1 and its downstream targets for the inhibition of lipid biosynthesis in tumor growth and progression of various cancers. This review could provide new insights into the critical function of the SREBP-1-regulated lipogenesis in different cancers and its potential for cancer therapy for targeting SREBP-1.

Critically, several issues and future perspectives are concerned, as in the following: 1) Targeting SREBP-1 can inhibit multiple enzymes in lipogenesis to significantly block cancer growth and aggressive progression, which is a promising strategy of multitarget therapeutics. 2) Current studies of targeting the SREBP-1 pathway against cancer are preclinical studies for investigating their effects and molecular mechanisms. It is necessary to conduct a clinical evaluation of SREBP-1-targeted therapy against cancer in future. 3) Except its role in cancer, SREBP-1 also plays a critical role in lipid metabolism of metabolic-related diseases. At present, no research is reported showing that those treatments targeting SREBP-1 have side effects, which might be related with the lower level of SREBP-1 in normal tissues, compared to that in cancer. The development of new and specific drugs targeting SREBP-1 with less or no toxic should be potential research directions.

## Author Contributions

QZ, XL, and GW: conceptualization, writing for original preparation. XL and GW: supervision. All authors have read and agreed to the published version of the manuscript.

## Funding

This study was supported by the National Natural Science Foundation of China (82100076). The funder had no role in the decision to publish or in the preparation of the manuscript.

## Conflict of Interest

The authors declare that the research was conducted in the absence of any commercial or financial relationships that could be construed as a potential conflict of interest.

## Publisher’s Note

All claims expressed in this article are solely those of the authors and do not necessarily represent those of their affiliated organizations, or those of the publisher, the editors and the reviewers. Any product that may be evaluated in this article, or claim that may be made by its manufacturer, is not guaranteed or endorsed by the publisher.

## Glossary

SREBPs: sterol regulatory element-binding proteins
bHLH-LZ: basic helix–loop–helix leucine zipper
GBM: glioblastoma
EGFR: epidermal growth factor receptor
PI3K: phosphatidylinositol 3-kinase
PKB: protein kinase B
mTOR: mammalian target of rapamycin
SCAP: SREBP cleavage-activating protein
SREs: sterol regulatory elements
ACLY: ATP citrate lyase
ACC: acetyl-CoA carboxylase
FASN: fatty acid synthase
SCD-1/5: stearoyl-CoA desaturase 1 and 5
LDLR: low-density lipoprotein receptor
mTORC1: mammalian/mechanistic target of rapamycin complex 1
GS: glutamine synthetase
O-GlcNAcylation: O-linked N-acetylglucosaminylation
Sp1: specificity protein 1
OGT: O-GlcNAc transferase
AMPK: AMP-activated protein kinase
sCLU: secretory clusterin
TDG: thymine DNA glycosylase
MAP: mitogen-activated protein
CRC: colorectal cancer
SNAIL: snail family transcriptional repressor 1
AR: androgen receptor
KLF5: Kruppel-like factor 5
HMGCR: hydroxy-3-methylglutaryl CoA reductase
GRP94: glucose-regulated protein 94
EGFRvIII: EGFR-activating mutation
HDGF: hepatoma-derived growth factor
AATF: apoptosis-antagonizing transcription factors
ZHX2: zinc fingers and homeoboxes 2
ACS: acyl-CoA synthetases
ACSL4: acyl CoA synthetase 4
SPIN1: spindlin 1
HBV: hepatitis B virus
HBx: hepatitis B virus-encoded X protein
STAT5: signal transducers and activators of transcription 5
GEO: Gene Expression Omnibus
TNM: tumor-node metastasis
PCK1: phosphoenolpyruvate carboxykinase 1
NSCLC: non-small cell lung cancer
PTMI5: protein arginine methyltransferase 5
DNFA: *de novo* fatty acid biosynthesis
SCCs: squamous cell carcinomas
TP63: tumor protein 63
ZEB1: zinc finger E-box-binding homeobox 1
LXRs: liver X receptors
PNKP: polynucleotide kinase/phosphatase
SNPs: single-nucleotide polymorphisms
SIRT1: sirtuin 1
FoxO1: forkhead transcription factor 1
ccRCC: clear cell renal cell carcinoma
E2F1: E2F transcription factor 1
EMT: epithelial–mesenchymal transition
NPC: nasopharyngeal carcinoma
GPX4: glutathione peroxidase 4
nSREBP-1: nuclear form of SREBP-1
PERK: protein kinase RNA-like endoplasmic reticulum kinase
ER: endoplasmic reticulum
PUFAs: polyunsaturated fatty acids
ATF6: activating transcription factor 6
S2P: site-2 protease
ERK1/2: endoplasmic reticulum kinase 1/2
FDFT1: farnesyl-diphosphate farnesyltransferase
APS: polysaccharides from *Astragalus membranaceus*.
